# Inhibition of androgen receptor enhanced the anticancer effects of everolimus through targeting glucose transporter 12

**DOI:** 10.7150/ijbs.75106

**Published:** 2023-01-01

**Authors:** Bo Cao, Ruiyang Zhao, Hanghang Li, Xingming Xu, Jingwang Gao, Lin Chen, Bo Wei

**Affiliations:** 1 Department of General Surgery, First Medical Center, Chinese PLA General Hospital, Beijing 100853, China.; 2 Medical School of Chinese PLA, Beijing 100853, China.

**Keywords:** Androgen receptor, Apoptosis, Everolimus, Gastric cancer, Glucose transporter 12

## Abstract

Everolimus was designed as a mammalian target of rapamycin (mTOR) inhibitor. It has been proven as a targeted drug for gastric cancer (GC) therapy. However, long-term treatment with everolimus may cause severe side effects for recipients. Decreasing the dosage and attenuating the associated risks are feasible to promote clinical translation of everolimus. This study aimed to identify the underlying mechanisms of responses to everolimus and develop novel regimens for GC treatment. Our findings proved that there was a significant dose-dependent relationship of everolimus-induced GC cell apoptosis and glycolysis inhibition. Then, we found that a member of glucose transporter (GLUT12) family, GLUT12, was actively upregulated to counteract the anticancer effects of everolimus. GLUT12 might be overexpressed in GC. High expression of GLUT12 might be correlated with tumor progression and short survival time of GC patients. Bioinformatic analysis suggested that GLUT12 might be involved in regulating cancer development and metabolism. The experiments proved that GLUT12 significantly promoted GC growth, glycolysis and impaired the anticancer effects of everolimus. Androgen receptor (AR) is a classical oncogenic factor in many types of cancer. Everolimus elevated GLUT12 expression in an AR-dependent manner. Inhibition of AR activity abrogated the promotive effects on GLUT12 expression. Both *in-vitro* and *in-vivo* experiments demonstrated that GLUT12 knockdown augmented anticancer effects of everolimus. Enzalutamide, an AR inhibitor, or AR knockdown was comparable to GLUT12 suppression. This study identified the role of the AR/GLUT12 pathway in the development of poor responses to everolimus. Interference with AR/GLUT12 pathway may serve as a promising approach to promoting the translational application of everolimus in GC therapy.

## Introduction

Gastric cancer (GC) is estimated to be one of the most threatening tumors worldwide. According to the latest epidemiological report, GC ranks the fifth for incidence and the fourth for cancer-associated mortality 1. Survival benefits from gastrectomy are limited for patients with GC at an advanced stage, due mainly to cancer metastasis and recurrence. The chemotherapeutic efficacy is also impaired by chemoresistance induced by long-term administration of drugs. It is urgent to develop novel regimens to enhance the shots and alleviate the side effects of targeted therapy. By virtue of understanding of carcinogenesis mechanisms, a battery of drugs targeting critical regulators of GC were invented and their efficacies were examined by clinical trials 2-4. Some of these drugs have already been included in alternative usages for clinical cancer treatment.

Everolimus is an orally bioavailable mammalian target of rapamycin (mTOR) inhibitor. Due to its potent effects on immunity suppression, everolimus has been applied for recipients of organ transplantation. mTOR is frequently abnormally expressed and mTOR signaling activation has been identified in cancer. Accumulating evidence has shown that mTOR is associated with malignant transformation, such as proliferation, invasion and stemness 5-7. Inhibition of mTOR activity is an effective approach to cancer therapy. Basic studies have reported that everolimus treatment induced apoptosis of cancer cells. Clinical trials have also verified the efficacies of everolimus in many types of cancers 8-10. However, the side effects set obstacles for further clinical application, such as infection, dental ulcer and metabolic disorders 11. Some studies have focused on the sensitization of cancer cells to everolimus with the aim of minimizing of the dosage and reducing adverse effects 12, 13. However, unmet needs of clinical therapy require more potential targets and accessible regimens.

Glucose transporter (GLUT) family is responsible for the first step of glucose uptake. Aberrant expression of GLUTs indicates poor prognosis and is required for cancer development. GLUT1, GLUT3 and GLUT4 have received the most attention 14-16. Inactivation of the targets has become a promising method of cancer treatment. GLUT12 was first identified in the MCF-7 cell line and proved to promote the progression of breast cancer 17, 18. Nevertheless, the functions of GLUT12 in GC remained unclear. Our study focused on the relationships between responses to everolimus and ectopic expression of GLUT12. The responses to everolimus are defined as GC suppression effects of specific doses of everolimus, embodied by cell apoptosis* in vitro* and tumor growth *in vivo* of this study. GLUT12 attenuated GC responses to everolimus under androgen receptor (AR)-dependent pathways. Inhibition of AR/GLUT12 axis plus everolimus treatment may become a promising therapeutic regimen for GC.

## Materials and methods

### Bioinformatic analysis of GLUT12 functions

The RNA-seq data of GC tissues in The Cancer Genome Atlas (TCGA) database were downloaded. The genes that were significantly correlated with GLUT12 expression were selected using Spearman analysis (*P*<0.05). The alterations of these selected genes may be mechanistically associated with GLUT12 functions. Gene Ontology (GO) and Kyoto Encyclopedia of Genes and Genomes (KEGG) analyses were then performed to enrich the biological functions of these genes. The enriched categories could suggest the regulatory role of GLUT12. The software R package operated by an online tool (https://www.xiantao.love) was employed to analyze the data and draw plots.

### Clinical specimens

To investigate the expression of GLUT12 in GC, a total of 60 patients who underwent gastrectomy at Chinese PLA General Hospital from May 2019 to March 2020 were enrolled. Patients who received chemotherapy, radiotherapy or targeted treatment were excluded from this study. GC and the corresponding adjacent tissues were immediately harvested after detachment. The lesions were diagnosed as gastric adenocarcinoma by two independent pathologists. Pathological features were determined according to the 8th cancer staging manual of American Joint Committee on Cancer. All the patients signed the informed consent. The use of clinical specimens was approved by Ethical Committee of Chinese PLA General Hospital.

### Cell culture

The cell lines used in this study were purchased from the American Type Culture Collection (Manassas, VA, USA) and cultivated in our laboratory. SGC-7901 cells labeled with luciferase (luc-SGC-7901) were specifically generated before this research. Cells were grown in Dulbecco's modified Eagle's medium (DMEM, Gibco; Thermo Fisher Scientific, MA, USA) supplemented with 10% fetal bovine serum (FBS, Kangyuan, Beijing, China) and 1% penicillin‑streptomycin (Gibco). The cultivation environment referred to the stationary atmosphere of 5% CO_2_ and 37 °C.

### Cell transfection and lentivirus package

Overexpression plasmids, small interfering RNAs (siRNAs) and short hairpin RNAs (shRNAs) were designed and synthesized by JTS Scientific (Wuhan, China). The sequences of siRNAs and shRNAs are displayed in Table S1. Lipofectamine 3000 (Thermo Fisher Scientific) was used for cell transfection. To generate cell lines with stable interference with targeted gene expression, the packaging vectors and specific interference plasmids were cotransfected into HEK-293T cells using the Lentiviral Packaging Kit (Yeasen, Shanghai, China). After 48 h, the virus was obtained and used to infect SGC-7901 and HGC-27 cells. 5 mg/mL puromycin was employed to chemically select cells with successful selection.

### Quantitative real-time PCR (qRT-PCR) analysis

Total RNA in cells and tissues was extracted with TRIzol Reagent (Invitrogen, NY, USA). Reverse transcription was conducted using the ExScript RT-PCR kit (TaKaRa, Japan) according to the protocol. Then, cDNA was amplified and marked with green fluorescence using SYBR Premix Ex Taq II (TaKaRa). Determination of β-actin expression was used for data calibration. The 2^-ΔΔCt^ method was employed to calculate the relative expression of targeted genes. The primers are listed in Table S2.

### Western blot (WB) analysis

Treated cells were harvested and washed with phosphate buffered saline (PBS) for three times. To obtain the protein samples, cells were suspended in chemical lysis buffer (Solarbio, Beijing, China) for 10 min to achieve cell lysis. The mixture underwent high-speed centrifugation and the supernatant was harvested. Protein concentration was determined using BCA Protein Assay Kit (Thermo Fisher Scientific). Total protein was separated by sodium dodecyl sulfate polyacrylamide gel electrophoresis and transferred onto polyvinylidene fluoride membranes (Millipore, MA, USA). After the exposure to blocking buffer for 1 h, the membranes were then incubated with primary antibodies at 4 ℃ overnight and anti-human antibodies at 25 ℃ for 1 h. ECL Western Blotting Substrate (Solarbio) was used to image the blots.

### Immunohistochemical (IHC) staining

To detect protein expression in clinical specimens, the tissues were washed three times with cold PBS to remove blood and impurities. Then, 4% paraformaldehyde was used to fix the proteins in tissues. IHC examination and score evaluation were performed as described in a previous study 19.

### Cell counting kit-8 (CCK-8) assay

To investigate cell proliferation, 3 × 10^3^ treated cells were seeded in 96-well plates. The medium was replaced by 100 μL DMEM. 10 μL of CCK-8 solution (Biorigin, Beijing, China) was prepared and gently added into the wells at the indicated times. The plates were placed in the incubator protected from light for 1 h. The absorbance at 450 nm was detected using a microplate reader (Biotek, VT, USA).

### Colony formation assay

Treated cells were suspended in complete DMEM and a total of 1 × 10^3^ cells were seeded in 6-well plates. They were grown in the incubator for 14 days. The medium was changed every 3 days. Cells were fixed with 4% paraformaldehyde for 10 min and stained with 0.1% crystal violet for 15 min. The colonies with over 50 cells were regarded as the positive count.

### 5-Ethynyl-2-deoxyuridine (EdU) assay

An EdU assay was also conducted to determine cell proliferation in this study. The 2 × 10^4^ treated cells were seeded in 96-well plates. Cells were labeled with EdU and stained with probes using a Cell Proliferation EdU Image Kit (Abbkine, Wuhan, China). Then the nuclei were stained with 4',6-diamidino-2-phenylindole (DAPI, Abbkine) to mark all cells in wells. The fluorescent pictures were imaged and recorded under a fluorescence microscope (Nikon, Japan).

### Flow cytometry of cell apoptosis

To detect the apoptosis induced by everolimus, cells were stained with apoptotic biomarkers using FITC Annexin V Apoptosis Detection Kit 1 (BD Pharmingen, NJ, USA). The data interpretation was conducted based on the intensity of Annexin V and propidium iodide (PI). Briefly, Annexin V- and PI-negative cells were markers of living cells. Cells labeled with Annexin V were at early apoptosis. Both positive Annexin V and PI served as hallmarks of late apoptosis.

### Glycolytic assays

Lactate acid, pyruvic acid, ATP production and glucose uptake can reflect glycolytic capability. Therefore, Lactate Colorimetric Assay Kit II, Pyruvate Colorimetric/Fluorometric Assay Kit, ATP Colorimetric/Fluorometric Assay Kit and Glucose Uptake Colorimetric Assay Kit (Biovision, CA, USA) were used. The prepared samples were transferred into 96-well plates, and the absorbances at the indicated wave lengths were measured by the microplate reader.

### Extracellular acidification rate (ECAR) and oxygen consumption rate (OCR) assays

A total of 1 × 10^4^ cells were seeded in a Seahorse XFe 96 Cell Culture Microplate (Agilent, USA). A Seahorse XF Glycolysis Stress Test Kit (Agilent, USA) was used to measure the ECAR. Glucose, oligomycin and 2-DG were sequentially added to the wells. For OCR, a Seahorse XF Cell Mito Stress Test Kit (Agilent) was employed, and cells were treated with oligomycin, FCCP and antimycin A/rotenone. Signals were read and recorded by a Seahorse XFe96 Analyzer (Agilent) at the indicated times.

### Animal experiment

Four-week-old male nude mice (Charles River, Beijing, China) were fed under the specific pathogen-free conditions. To generate cell-derived xenografts, mice were randomly divided into groups. A total of 5 × 10^6^ luc-SGC-7901 cells were injected subcutaneously into the mouse right flank subcutaneously. After 7 days, mice were treated with everolimus. The lengths and widths were measured using the vernier caliper at intervals of 7 days. Volume = length × width^2^/2. After 30 days, 100 μL of 15 mg/mL D-luciferin (Solarbio) was intraperitoneally injected into mice. Tumor load was detected using the *In-Vivo* Imaging System (PerkinElmer, USA). Mice were sacrificed by CO_2_ asphyxia. To investigate the side effects of the therapeutic regimens, we chose weight as the indicator. Nude mice without GC transplantation received the same interventions. Mouse weights were measured by an electronic scale at the indicated time. The animal experiments were approved by Animal Center of Chinese PLA General Hospital. Animal experiments conformed to the Animal Research: Reporting of *In Vivo* Experiments (ARRIVE) guidelines.

### Statistical analysis

SPSS 25.0 was used to conduct statistical analysis. Prism 8.0 was employed to draw diagrams in figures. Survival analysis was performed with the support of the online bioinformatic tool Kaplan-Meier Plotter 20. Data were presented as mean ± standard deviation (SD). Comparison of variables between groups was conducted using two-sided Student's *t* test and one-way ANOVA. *P <* 0.05 was regarded as the significant difference. The experiments were performed in triplicates and repeated at least three times.

## Results

### Everolimus induces apoptosis of GC in a dose-dependent manner

**Figure 1:**
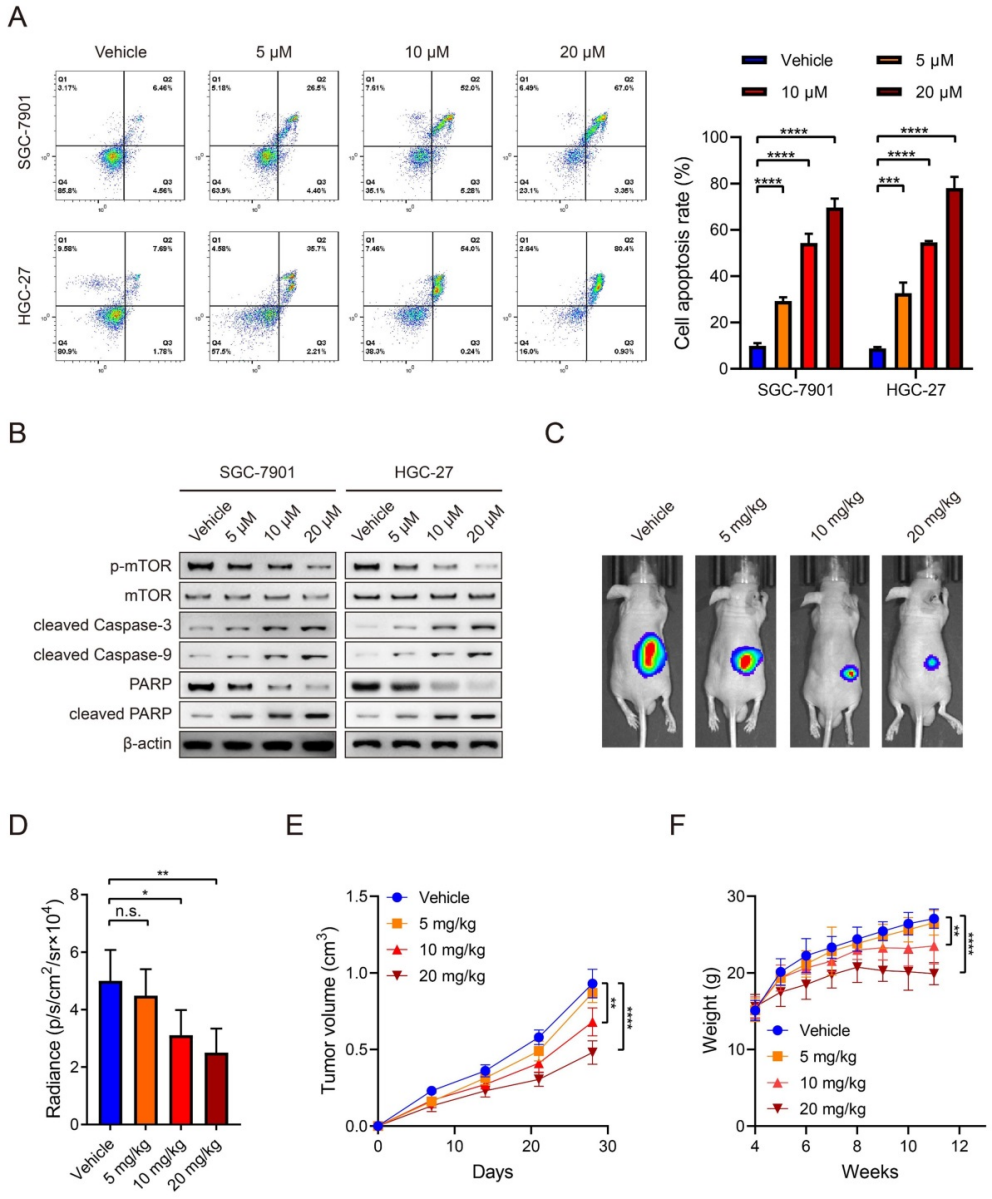
Everolimus induces apoptosis of GC in a dose-dependent manner.** A** The flow cytometric analysis to show the apoptosis of SGC-7901 and HGC-27 cells that were treated with vehicle or 5 μM, 10 μM or 20 μM of everolimus. Histograms are on the right. **B** The WB analysis to show the protein expression of cells as in (**A**). **C** The representative bioluminescence images of nude mice after 30 days of subcutaneous injection of luc-SGC-7901 cells. Nude mice were intraperitoneally injected with vehicle or 5mg/kg, 10 mg/kg or 20 mg/kg of everolimus daily. **D** The luminescence signal intensities of nude mice as in (**C**). **E** The curve of tumor volumes as in (**C**) at the indicated time. **F** The weight of nude mice as in (**C**) at the indicated time. *P<0.05, **P<0.01, ***P<0.001, ****P<0.0001, n.s. no significant.

Everolimus could effectively induce apoptosis in SGC-7901 and HGC-27 cell lines and there was a positive dose-dependent relationship (Figure 1A). Regarding the role of everolimus as an mTOR inhibitor, WB analysis was used to examine its inhibitory effects on mTOR activity. The results demonstrated that everolimus significantly inhibited mTOR phosphorylation, which is consistent with a previous study 21. The expression of cleaved caspase-3 and caspase-9, two kinds of apoptotic markers, was elevated with increasing doses of everolimus. PARP expression was reduced while cleaved PARP was inversely increased (Figure 1B). To further evaluate the potential value of everolimus in GC therapy, we generated nude mice with subcutaneous tumors. They received intraperitoneal administration of vehicle or 5 mg/kg, 10 mg/kg or 20 mg/kg of everolimus, respectively. After 30 days, everolimus also inhibited tumor growth *in vivo* in the dose-dependent manner (Figure 1C-E). However, we found that middle- and high-dose usage of everolimus led to significant weight loss in mice (Figure 1F). These data reveal that everolimus induces apoptosis and inhibits tumor growth. Long-term administration of large doses may cause severe side effects of recipients.

### Everolimus treatment reprograms glycolysis and enhances GLUT12 expression

**Figure 2:**
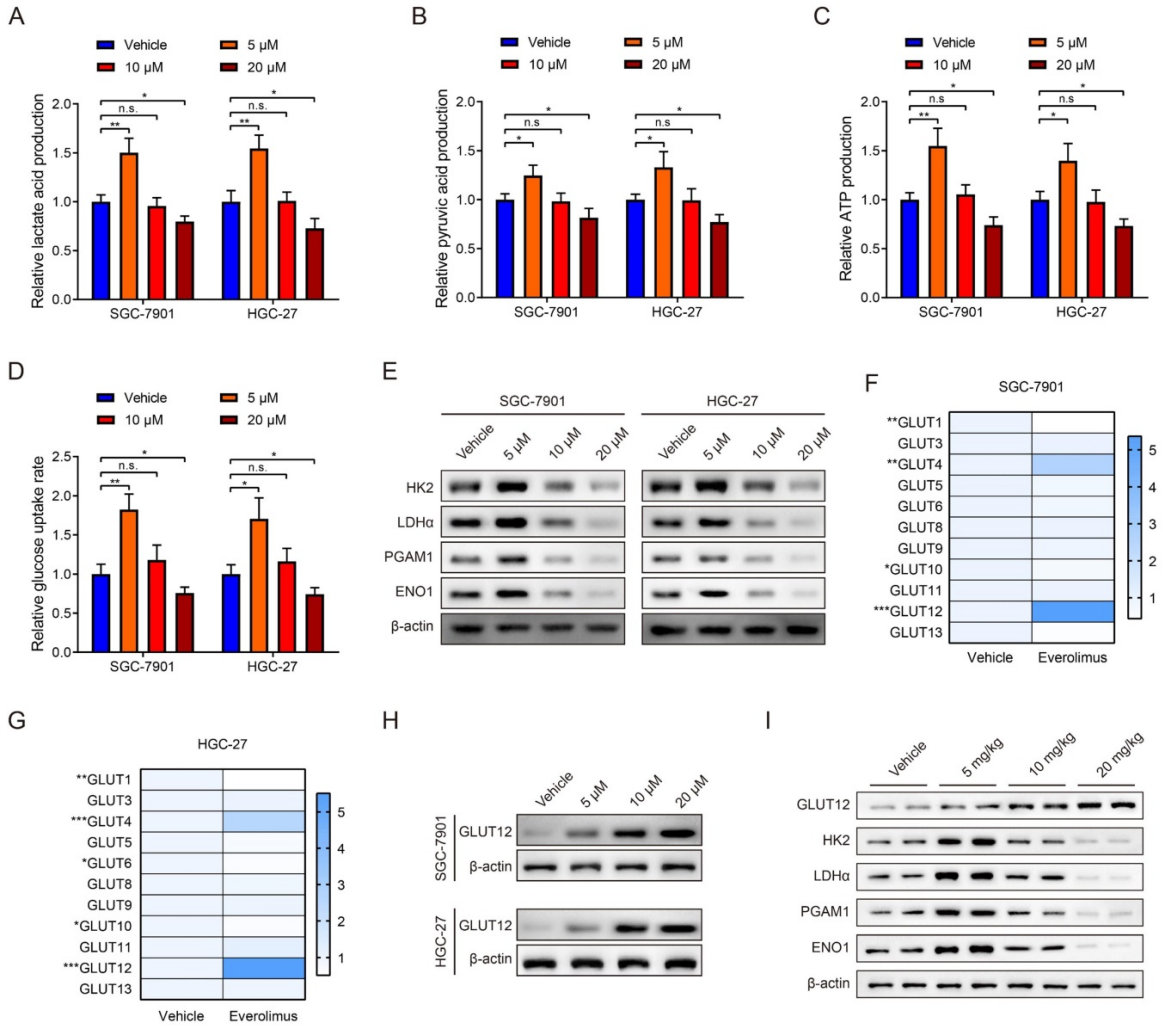
Everolimus treatment reprograms glycolysis and enhances GLUT12 expression.** A-D** The colorimetric assays to determine the production of lactate acid (**A**), pyruvic acid (**B**), ATP production (**C**) and relative glucose uptake rates (**D**) of SGC-7901 and HGC-27 cells that were treated with vehicle or 5 μM, 10 μM or 20 μM of everolimus. **E** The WB analysis to show the protein expression of cells as in (**A-D**). **F**, **G** The heatmap to display the mRNA expression of GLUTs in SGC-7901 (**F**) and HGC-27 cells (**G**) that were treated with vehicle or 5 μM of everolimus. **H** WB analysis to show the GLUT12 expression of cells as in (**A-D**). **I** WB analysis to show the protein expression in tumor tissues collected from nude mice that were intraperitoneally injected with vehicle or 5mg/kg, 10 mg/kg or 20 mg/kg of everolimus daily. *P<0.05, **P<0.01, ***P<0.001, n.s. no significant.

mTOR activation is closely associated with glycolysis in cancer 22, 23. Therefore, we explored the effects of everolimus treatment on glycolysis. The levels of lactate acid, pyruvic acid, ATP production and glucose uptake rates in GC cells were determined after everolimus treatment. Surprisingly, the results showed that a low dose of everolimus moderately increased lactate acid, pyruvic acid, ATP production and rates of glucose uptake. The usage of middle- and high-dose everolimus conversely reduced the four indicators of glycolysis (Figure 2A-D). WB analysis was used to detect alterations in glycolysis-associated proteins, including hexokinase 2 (HK2), lactate dehydrogenase α (LDHα), phosphoglycerate mutase 1 (PGAM1) and enolase 1 (ENO1). Similarly, low-dose everolimus elevated their expression, and they were inhibited with the increasing doses (Figure 2E). This unexpected phenomenon suggested underlying mechanisms of poor responses to everolimus through glycolysis potentiation. Because of the mechanistic associations between drug responses and ectopic expression of GLUTs in cancer 24, 25, we measured 14 members of the GLUT family in GC cells treated with the low dose of everolimus. GLUT2, GLUT7 and GLUT14 were undetected. Among the remaining 11 GLUTs, GLUT12 was the most overexpressed in both SGC-7901 and HGC-27 cells (Figure 2F, G). WB analysis also confirmed the increased GLUT12 expression induced by everolimus (Figure 2H). Furthermore, we detected expression of GLUT12 and glycolysis-associated proteins in the subcutaneous tumors collected from mice treated with different doses of everolimus. The results confirmed that GLUT12 protein expression was positively correlated with doses of everolimus. Low-dose everolimus could moderately enhance GC glycolysis, while increasing doses led to significant inhibition of glycolysis (Figure 2I).

### Identification of GLUT12 clinical significance in GC

Because of the unknown role of GLUT12 in GC, we first investigated its clinical significance. Clinical information from two Gene Expression Omnibus (GEO) datasets (GSE62254 and GSE15459) was analyzed. In the GSE62254 dataset, the overall survival (OS) of patients in the low-expression group was mildly better than the OS of patients in the high-expression group without significance (Hazard Ratio (HR) = 1.25, 95% confidence interval (CI) 0.84-1.85, *P* = 0.28, Figure S1A). However, the progression-free survival (PFS) in the low-expression group was significantly higher than the PFS in the high-expression group (HR = 1.61, 95% CI 1.1-2.35, *P* = 0.012, Figure S1B). The analytic results from the GSE15459 dataset were similar. No significant difference was observed in OS (HR = 1.47, 95% CI 0.99-2.18, *P* = 0.055, Figure S1C), while GLUT12 expression could differentiate PFS between the high-expression and low-expression groups (HR = 1.53, 95% CI 1.06-2.21, *P* = 0.021, Figure S1D). We also performed survival analysis based on patients collected from TCGA database. There was no difference of OS between GLUT12 high- and low-expression groups (HR = 0.99, 95% CI 0.72-1.38, *P* = 0.969, Figure S1E). High expression of GLUT12 was identified in GC tissues using qRT-PCR and IHC analysis (Figure S1F-H). GLUT12 expression was also positively correlated with the T and TNM stage (Table 1). These data suggest the overexpression state of GLUT12 in GC tissues and it serves as a prognostic biomarker for GC patients.

### Enrichment analysis of GLUT12 functions in GC

**Figure 3:**
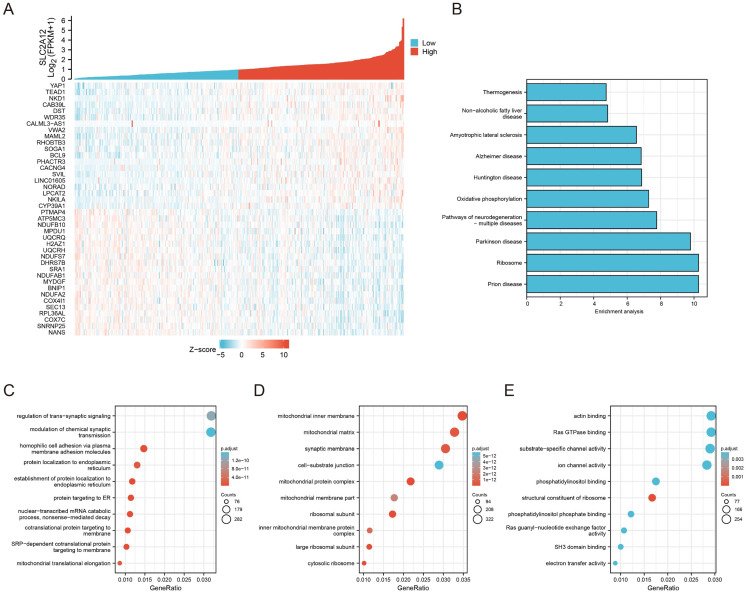
Enrichment analysis of GLUT12 functions in GC.** A** The relative expression of GLUT12 in GC tissues from TCGA database was shown above. Patients were divided into low and high groups according to the median expression. Top 20 genes that are the most positively associated with GLUT12 expression and top 20 genes that are the most negatively associated with it were displayed below. **B** The histogram to display top 10 pathways that are associated with GLUT12 expression analyzed by KEGG. **C** Top 10 BP terms that are significantly enriched in GO analysis. **D** Top 10 CC terms that are significantly enriched in GO analysis. **E** Top 10 MF terms that are significantly enriched in GO analysis.

The RNA-seq data of 407 GC specimens were downloaded from the TCGA database. Genes with expression significantly correlated with GLUT12 expression (*P <* 0.05) were screened out. The specimens were divided into low and high groups based on the median value of GLUT12 expression. The heatmap displayed top 40 genes of them (Figure 3A). The genes were enriched by KEGG and GO analysis. As shown by KEGG, oxidative phosphorylation was enriched in the top 10 categories. The imbalance between glycolysis and oxidative phosphorylation has been regarded as a hallmark of carcinogenesis and progression 26. The categories concerning neurological disorders, including Prion disease, Parkinson disease, Pathways of neurodegeneration-multiple diseases, Huntington disease, Alzheimer disease and Amyotrophic lateral sclerosis, were listed in the top 10 pathways. Loss of normal metabolic modulation has been regarded as a critical reason of these diseases 27-29. Moreover, non-alcoholic fatty liver disease and thermogenesis were also enriched among the top 10 pathways, which are also closely associated with glycolysis dysregulation 30, 31. Hence, the data of KEGG analysis suggest that GLUT12 may participate in the regulation of GC glycolysis (Figure 3B).

GO analysis can be divided into three parts based on gene functions, including biological process (BP), cellular component (CC) and molecular function (MF). Most of the top 10 categories in BP analysis were associated with cell membrane structures (Figure 3C). The CC categories were majoring in mitochondrial functions (Figure 3D). The MF categories were also associated with membrane functions (Figure 3E). The abnormal functions of membrane have been revealed as a critical contributor of malignant proliferation 32-34, which also confirmed the data credibility due to the close relationships between GLUT12 and cell membrane. The mitochondrial function is also an important indicator of glycolysis 35. Taken together, the bioinformatic analysis suggests that GLUT12 may regulate oncological behaviors and metabolism of GC.

### GLUT12 overexpression promotes GC proliferation and attenuates responses to everolimus

**Figure 4:**
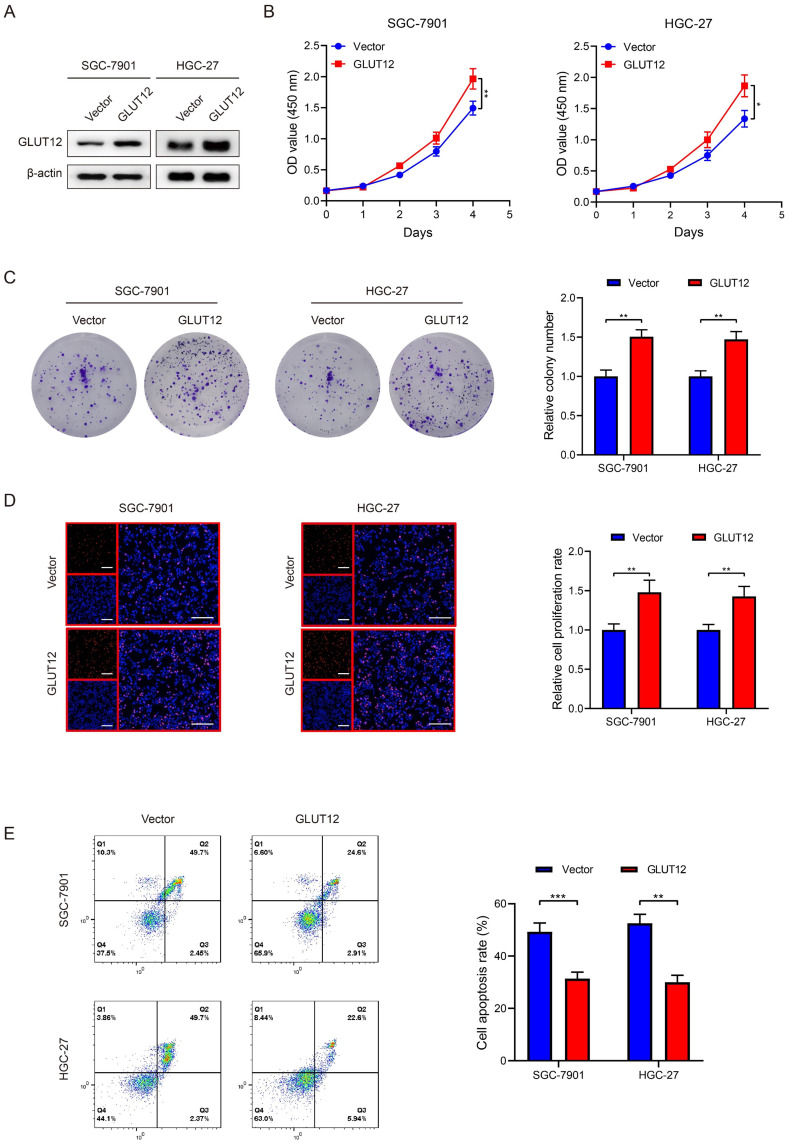
GLUT12 overexpression promotes GC proliferation and attenuates responses to everolimus.** A** The WB analysis to show GLUT12 expression of SGC-7901 and HGC-27 cells that were infected with lentivirus carrying vectors or GLUT12 overexpression plasmids. **B-D** The CCK-8 (**B**), colony formation (**C**) and EdU (**D**) assays to measure proliferation of cells as in (**A**). Histograms of colony formation and EdU data are on the right. Scale bar: 100 μm. **E** The flow cytometric analysis to show the apoptosis of cells as in (**A**) that were treated with 10 μM of everolimus. Histograms are on the right. *P<0.05, **P<0.01, ***P<0.001.

Regarding the close relationships between GLUT12 and cancer proliferation, we established SGC-7901 and HGC-27 cells that stably overexpressed GLUT12, which was validated by WB analysis (Figure 4A). CCK-8, colony formation and EdU assays demonstrated that GLUT12 expression upregulation promoted the proliferation of SGC-7901 and HGC-27 cells (Figure 4B-D). We speculated that upregulation of GLUT12 expression could protect GC cells from apoptosis induced by everolimus. The results showed that apoptotic rates were reduced in cells overexpressing GLUT12 (Figure 4E). Next, SGC-7901 and HGC-27 cells with stable knockdown of GLUT12 were also generated to explore the effects of GLUT12 expression downregulation on GC malignancy (Figure S2A). Only slight decreases in cell proliferation without significance were observed (Figure S2B-D). The responses to everolimus were also insignificantly changed (Figure S2E). Our findings prove that overexpression of GLUT12 promotes GC proliferation and reduces responses to everolimus. However, GLUT12 knockdown cannot suppress these malignant behaviors.

To further reaffirm the results of enrichment analysis based on TCGA database, we chose the top 3 genes positively correlated with GLUT12 expression, including Yes-associated protein 1 (YAP1), TEA domain family member 1 (TEAD1) and NKD Inhibitor Of WNT Signaling Pathway 1 (NKD-1), and top 3 genes negatively correlated with GLUT12 expression, including N-Acetylneuraminate Synthase (NANS), Small Nuclear Ribonucleoprotein U11/U12 Subunit 25 (SNRNP25) and Cytochrome C Oxidase Subunit 7C (COX7C), for expression determination using qRT-PCR. GLUT12 overexpression significantly upregulated expression of YAP1, TEAD1 and NKD1 and downregulated expression of NANS, SNRNP25 and COX7C both in SGC-7901 and HGC-27 cells. For the results of knockdown of GLUT12, the expression of YAP1, TEAD1 and NKD1 was decreased and the levels of NANS and SNRNP25 expression were elevated. However, COX7C expression was mildly upregulated in cells with stable knockdown of GLUT12 (Figure S3A-F). The data were roughly consistent with TCGA database, suggesting the reliability of bioinformatic analysis for identification of GLUT12 functions in GC.

### Upregulation of GLUT12 expression reprograms GC glycolysis

**Figure 5:**
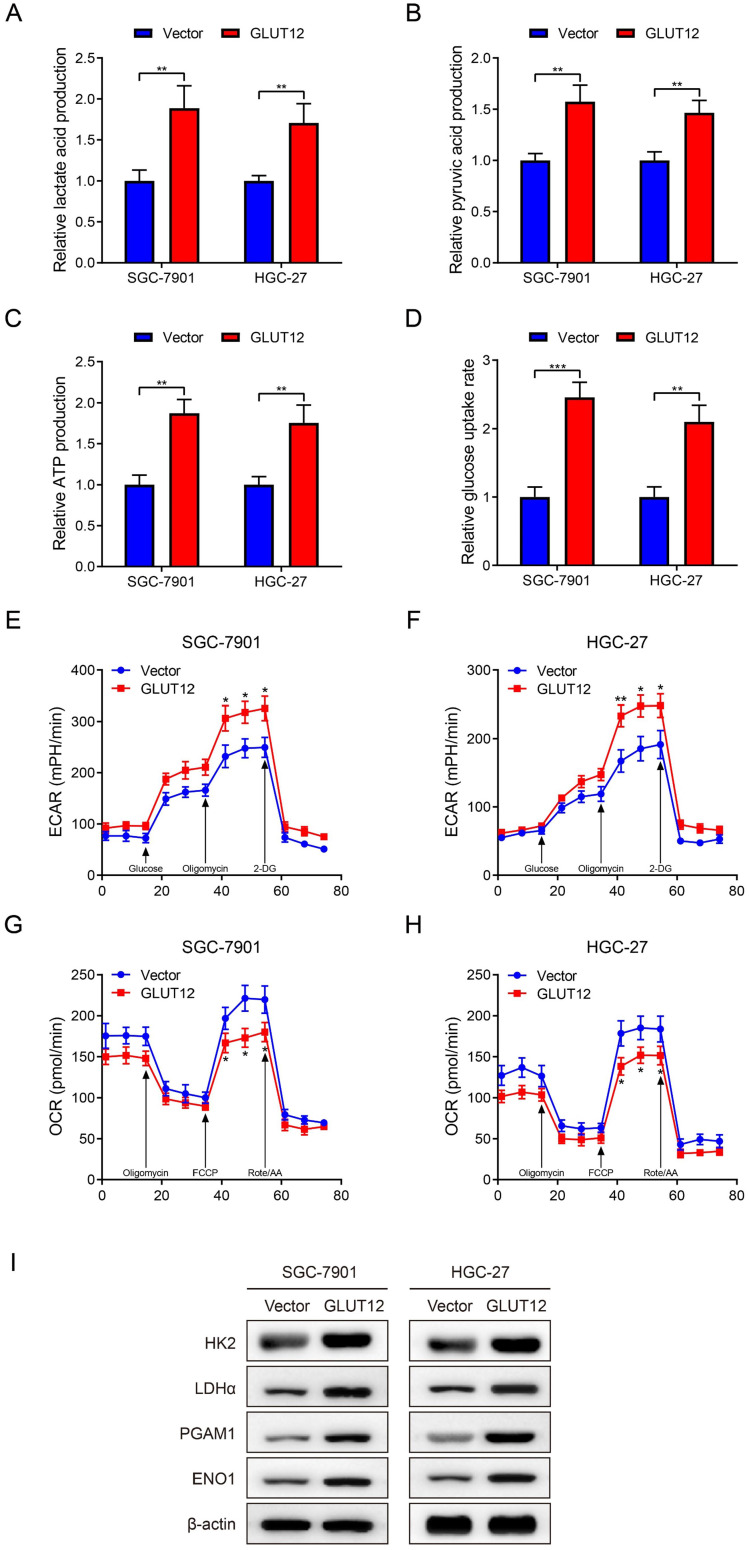
Upregulation of GLUT12 expression reprograms GC glycolysis.** A-D** The colorimetric assays to determine the production of lactate acid (**A**), pyruvic acid (**B**), ATP production (**C**) and relative glucose uptake rates (**D**) of SGC-7901 and HGC-27 cells that were infected with lentivirus carrying vectors or GLUT12 overexpression plasmids. **E**, **F** ECAR assay to show glycolysis levels of cells as in (**A-D**). **G**, **H** OCR assay to show oxidative phosphorylation levels of cells as in (**A-D**). **I** The WB analysis to show protein expression of cells as in (**A-D**). *P<0.05, **P<0.01, ***P<0.001.

GLUT12 is a member of the GLUT family and regulates glucose uptake, and GLUT12 may be associated with GC glycolysis based on the bioinformatic data. Thus, glycolytic experiments were used to detect glycolysis after GLUT12 overexpression. Upregulation of GLUT12 expression promoted lactate acid, pyruvic acid, ATP production and rates of glucose uptake (Figure 5A-D). The ECAR and OCR reflect the levels of glycolysis and oxidative phosphorylation, respectively. ECAR was enhanced in cells overexpressing GLUT12 (Figure 5E, F). OCR was inversely suppressed in SGC-7901 and HGC-27 cells (Figure 5G, H). This imbalance indicated a greater dependence on glycolysis induced by GLUT12 overexpression. WB analysis indicated that elevated GLUT12 expression increased HK2, LDHα, PGAM1 and ENO1 expression (Figure 5I). Furthermore, the glycolytic experiments with additional treatment of everolimus showed that knockdown of GLUT12 could achieve conversion from mild glycolysis enhancement to significant suppression induced by low-dose everolimus (Figure S4A-E). The results prove that GLUT12 overexpression enhances glycolysis and maintains relatively low responses to everolimus of GC cells.

### AR mediates poor responses to everolimus through upregulating GLUT12 expression

**Figure 6:**
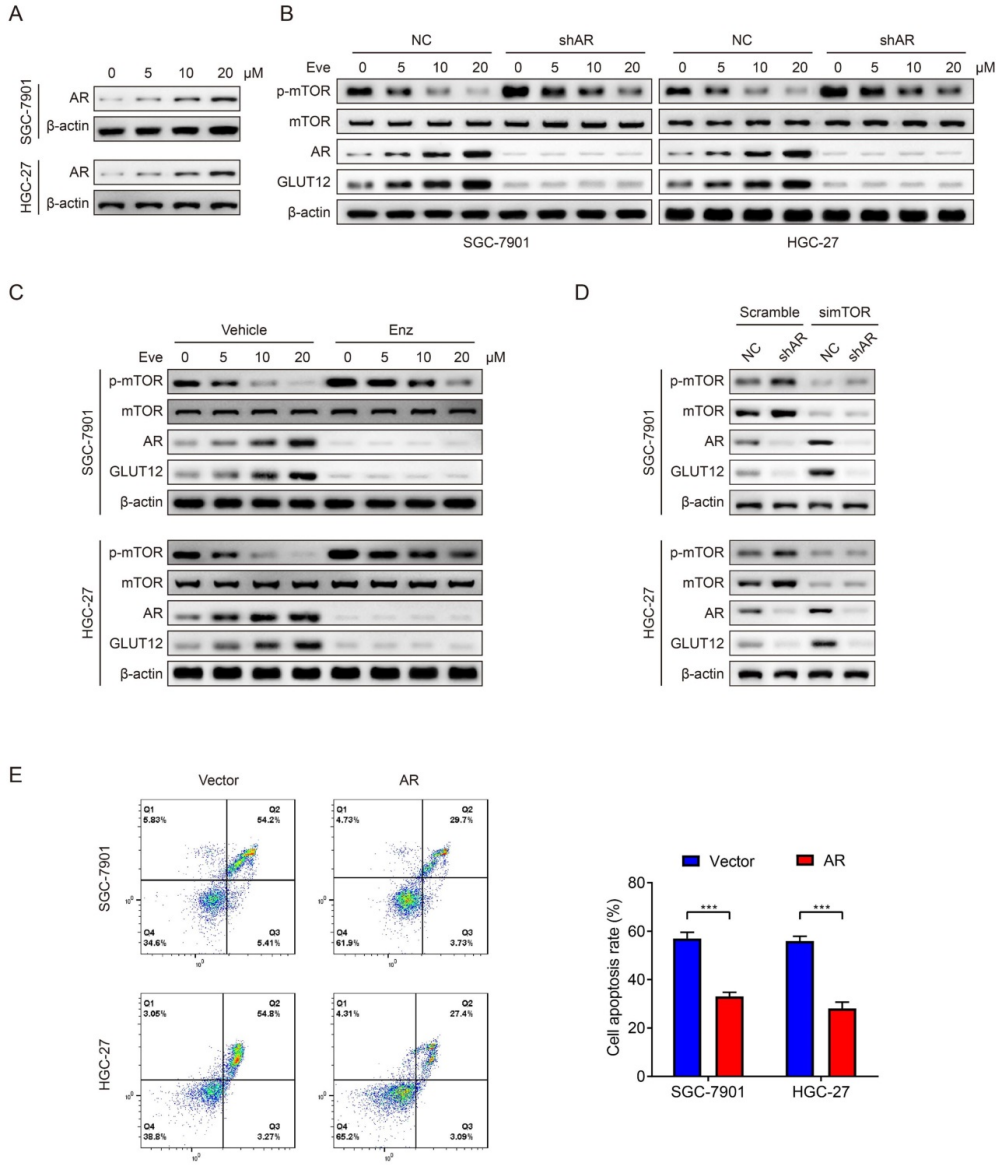
Everolimus induces GLUT12 overexpression by upregulating AR levels.** A** The WB analysis to show AR expression of SGC-7901 and HGC-27 cells that were treated with vehicle or 5 μM, 10 μM or 20 μM of everolimus. **B** The WB analysis to show protein expression of control or AR-knockdown cells that were treated with vehicle or 5 μM, 10 μM or 20 μM of everolimus, respectively. **C** The WB analysis to show protein expression of cells that were treated with vehicle or 25 μM of enzalutamide and vehicle or 5 μM, 10 μM or 20 μM of everolimus, respectively. **D** The WB analysis to show protein expression of control or AR-knockdown cells that were transfected with scramble siRNA or siRNA targeting mTOR. **E** The flow cytometric analysis to show the apoptosis of cells that were infected with lentivirus carrying vectors or AR overexpression plasmids with treatment of 10 μM of everolimus. Histograms are on the right. ***P<0.001.

Mark et al reported that GLUT12 was regulated by the AR pathway 36. Therefore, we speculated that GC cells upregulated GLUT12 expression through AR pathways to resist against everolimus. In consideration of relatively low AR expression in GC as previously reported 37, we chose SNU-1 cell line as a negative control and LNCaP cell line as a positive control. The detectable AR expression in SGC-7901 and HGC-27 cells was confirmed by WB analysis (Figure S5). Next, the results showed that AR expression was elevated after everolimus treatment, which was positively correlated with drug concentrations (Figure 6A). Cells with stable knockdown of AR were successfully constructed. Downregulation of AR expression activated mTOR phosphorylation. It also abrogated the effects of everolimus on GLUT12 expression upregulation (Figure 6B). We then used an AR antagonist, enzalutamide, to treat cells. Enzalutamide had the effects on mTOR and GLUT12 regulation similar to AR shRNA (Figure 6C). To exclude the off-target effects of everolimus, we designed an siRNA targeting mTOR. AR knockdown also abolished the promotion of GLUT12 expression by mTOR inhibition (Figure 6D). These results validate the participation of AR/GLUT12 axis in reliving responses to everolimus in GC.

Regarding the close associations between AR and GLUT12, it was speculated that AR might also serve as an oncogenic factor. To validate this assumption, we generated SGC-7901 and HGC-27 cell lines with stable overexpression of AR (Figure S6A). Upregulation of AR expression promoted GC proliferation proved by CCK-8, colony formation and EdU assays (Figure S6B-D). AR overexpression also led to a significant decrease in apoptotic rates induced by everolimus (Figure 6E). The abovementioned findings suggest that AR serves as a regulator of malignant proliferation and responses to everolimus.

### Suppression of the AR/GLUT12 pathway synergistically enhanced the anticancer effects of everolimus

**Figure 7:**
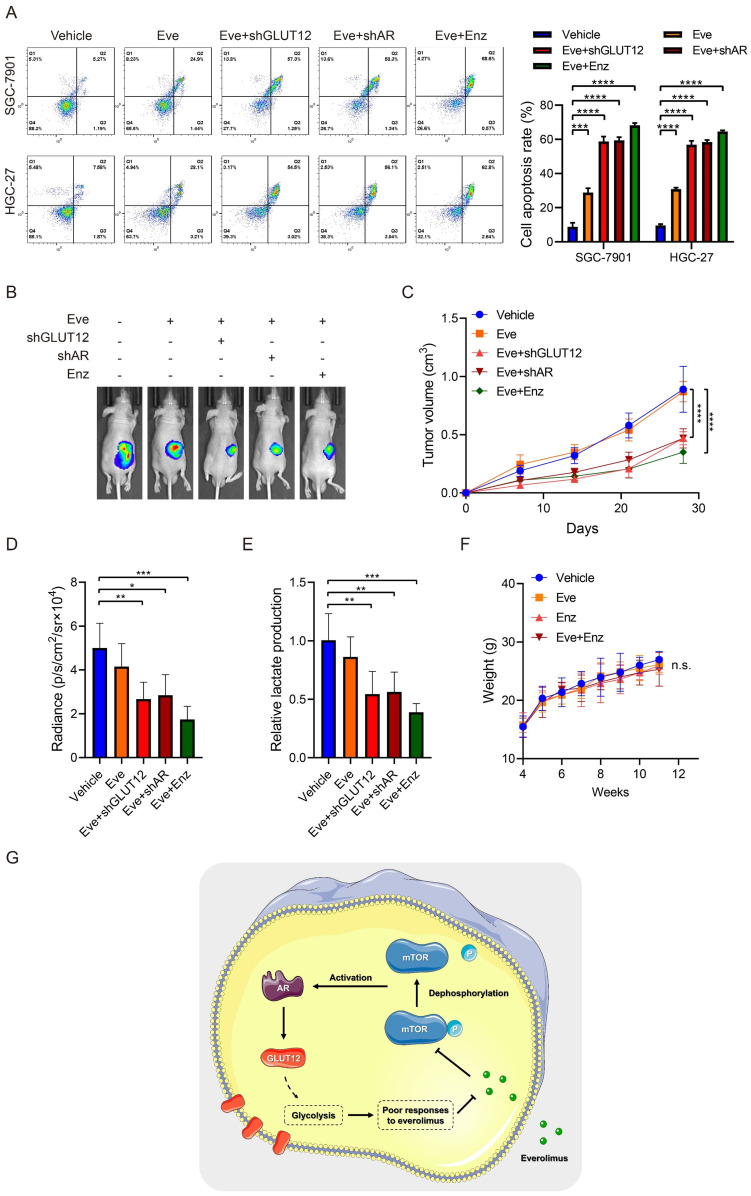
Suppression of the AR/GLUT12 pathway synergistically enhanced the anticancer effects of everolimus.** A** The flow cytometric analysis to show the apoptosis of SGC-7901 and HGC-27 cells with control shRNA, GLUT12 knockdown or AR knockdown that were treated with vehicle or 5 μM everolimus or treated with vehicle or 25 μM enzalutamide. Histograms are on the right.** B** The representative bioluminescence images of nude mice after 30 days of subcutaneous injection of luc-SGC-7901 cells with control shRNA, GLUT12 knockdown or AR knockdown. Nude mice were treated with vehicle, 5 mg/kg of everolimus or 10 mg/kg of enzalutamide daily, respectively. **C** The curve of tumor volumes as in (**B**) at the indicated time. **D** The luminescence signal intensities of nude mice as in (**B**). **E** The colorimetric assay to determine the production of lactate acid as in (**B**).** F** The weight change of nude mice at the indicated time. **G** Schematic illustration of mechanisms underlying AR/GLUT12 pathway in regulating everolimus resistance. *P<0.05, **P<0.01, ***P<0.001, ****P<0.0001. n.s. no significant.

Since the important role of the AR/GLUT12 axis in the impairment of responses to everolimus, GLUT12 or AR suppression was assumed to possibly enhance the efficacies of everolimus. Apoptosis assays demonstrated that cell apoptosis was elevated in cells with GLUT12 or AR knockdown or enzalutamide treatment compared to the single everolimus treatment (Figure 7A). Next, we examined the inhibitory effects of these combinational regimens on glycolytic reprogramming. GLUT12 knockdown and AR inhibition served as glycolysis suppressors under everolimus treatment. There were no significant differences between the three additional interventions (Figure S7A-E).

Xenografts were generated to validate the effectiveness of this regimen. Single treatment with everolimus moderately inhibited tumor growth. GLUT12 knockdown augmented the inhibitory effects of everolimus. Blocking AR functions also exhibited significant efficacies of tumor growth suppression (Figure 7B-D). The lactate acid was determined in tumor tissues. The results were also consistent with *in-vitro* glycolytic experiments. Both GLUT12 and AR inhibition suppressed lactate acid production in tumors (Figure 7E). Moreover, there was insignificant weight loss when mice were the drugs (Figure 7F). Collectively, our findings proved that GC cells actively activated AR/GLUT12 pathways to resist against everolimus treatment. Blocking AR functions could sensitize cells to everolimus without severe side effects (Figure 7G).

## Discussion

mTOR is a serine/threonine kinase that obtains inputs from nutrients, growth factors and environmental cues. mTOR regulates multiple physiological and pathological processes, including metabolism, biosynthesis, development, aging, inflammation and immunity 38. Everolimus was initially designed as an mTOR inhibitor for immunosuppression. Dysregulation of mTOR activation during carcinogenesis was identified, and its underlying mechanisms as an oncogenic factor were elucidated by existing studies 39. The potential value of everolimus in cancer treatment has also been validated by basic experiments and clinical trials 8-10. Everolimus was approved by the Food and Drug Administration as an alternative drug against metastatic renal carcinoma, advanced pancreatic neuroendocrine tumors and subependymal giant cell astrocytoma. However, some reports claimed concerns about drug insensitivity and side effects induced by long-term usage of everolimus 40, 41. The debates on survival benefits from everolimus treatment still confuse clinicians 42. The weight loss of nude mice treated with everolimus in this study also suggested that the side effects cannot be neglected. It is urgently required to explore effective approaches to sensitization of cancer cells and decreases in the administered doses in clinical practice.

To the best of our knowledge, this is the first study to report the role of GLUT12 in GC progression. Only a few studies have focused on the biological functions of GLUT12 since its first identification in 2002 17. It was reported to regulate multiple physiological and pathological processes, including oocyte development, polycystic ovarian syndrome and obesity 43-45. For carcinogenesis, Matsui et al found that GLUT12 overexpression promoted breast cancer migration 46. Shi et al elucidated the critical functions of the let-7a-5p/GLUT12 axis in the malignant progression of triple-negative breast cancer 18. We speculated that GLUT12 acted as a regulator of drug responses due to its ectopic upregulation after everolimus treatment. Bioinformatic and experimental data were conjointly obtained to verify our assumption. GLUT12 had potentials in the prediction of GC progression and patient survival. Upregulation of GLUT12 expression promoted proliferation and glycolysis and protected GC cells from apoptosis induced by everolimus. Nevertheless, GLUT12 knockdown did not significantly inhibit GC progression, which is contrary to the previous findings 18, 36, 46. This inconsistency may be attributable to the low-expression background or 'stand-by' state of GLUT12 without everolimus treatment in GC. Moreover, we employed GEO and TCGA datasets to analyze the prognostic value of GLUT12, whereas the results based on different cohorts are distinct. Similar results of other molecules have been observed in our previous studies 47. The discrepancy may be, at least partly, attributable to cutoff points, experimental methods of gene expression detection, different included populations and some unknown potential bias. The translational value of GLUT12 in prognostic evaluation requires further investigations based on large-sample clinical trials. It also suggests that single biomarker may be incompetent in accurately predicting patient survival and molecular combination is a promising research direction.

Mark et al reported that AR directly regulated GLUT12 expression by directly binding to an intronic region of *SLC2A12*. This evidence hinted us that GLUT12 might be also regulated by AR in GC. The data demonstrated that everolimus-induced GLUT12 overexpression was abrogated by knockdown of AR. AR is emerging as a potential target of cancer therapy. Most studies have focused on its functions in prostate cancer, breast cancer and hepatocellular carcinoma due to the specific distribution in these organs. Recently, AR has been proven to be a prognostic biomarker and therapeutic target of GC. Combinational evaluation of EGFR and AR expression could predict prognosis of GC patients 48. Moreover, AR may be correlated with Lauren classification, suggesting its potentials in assisting tumor classification in clinical practice 49, 50. Upregulation of AR expression have also been found to promote GC proliferation, migration and invasion 51. The mechanistic associations between AR/GLUT12 axis and poor responses to everolimus were first identified by our findings, suggesting that AR may serve as a promising target for clinical GC therapy.

The mechanisms underlying cancer self-rescue under the conditions of drug treatment become a hot research topic. Cancer cells can mobilize their potential and activate underlying mechanisms to adapt to harsh environments and ameliorate the anticancer effects of drugs. For instance, breast cancer has been reported to upregulate Rac1 expression to resist against chemotherapeutic-induced DNA damage 52. SOX2-induced CD24 expression upregulation can confer adaptive resistance toward BRAF inhibitors in melanoma 53. The protective functions of glycolysis activation in responses to anticancer drugs have also been revealed 54-56. In the present study, the middle and high doses of everolimus suppressed glycolysis, which is consistent with the results of mTOR inhibition reported by other studies 23, 57. However, the low-dose treatment conversely enhanced GC glycolysis. This contradiction may be attributable to the counteractive effects between mTOR signaling and AR/GLUT12 axis. The inhibition of mTOR activity by low-dose everolimus did not effectively suppress glycolysis, whereas AR/GLUT12 axis was activated and enhanced GC glycolysis. Naturally, the moderate upregulation of glycolytic levels was observed in cells treated with low-dose everolimus. The phenotype bounce using low-dose drug has also been reported by other studies 47. The evidence suggests that the mechanisms underlying cancer self-rescue should be deeply investigated. Development of effective regimens to overcome it may be a novel direction for future research.

Regarding the important role of the AR/GLUT12 pathway in GC, we speculated that inhibition of AR or GLUT12 could enhance the efficacies of everolimus. Both *in-vitro* and *in-vivo* experiments verified our assumption. AR/GLUT12 axis inhibition plus enzalutamide treatment also significantly attenuated glycolysis compared to administration of everolimus alone. The insignificant weight loss preliminarily proved the safety of the regimens. These results jointly suggest that AR/GLUT12 axis can participate in the maintenance of low responses to everolimus. Notably, the effects of enzalutamide treatment were comparable to GLUT12 or AR knockdown in suppressing GC growth, which provide a feasible approach to clinical GC therapy due to the lack of GLUT12 inhibitors currently.

We have to admit that our research has some limitations. First, we found that everolimus induced activation of the AR/GLUT12 axis. The specific mechanisms by which AR regulates GLUT12 expression need further exploration. Second, few studies have focused on the enhancement of responses to everolimus. In addition to GLUT12, there may be more effective targets. More studies, especially high-throughput screening, should be conducted to identify mechanistic networks of targeted therapy responses. Third, the optimal usage and effectiveness for clinical application need more investigation. Fourth, mTOR signaling pathway can affect various cellular phenotypes, not limited to glycolysis. The other underlying mechanisms of everolimus should be deeply investigated.

In summary, our findings identified high expression of GLUT12 in GC. GLUT12 expression in tissues was associated with cancer progression and poor survival time of GC patients. GLUT12 overexpression could promote GC proliferation. Importantly, GC cells actively upregulated GLUT12 to relieve anticancer effects of everolimus through facilitating AR pathway. Inhibition of the AR/GLUT12 pathway enhanced the anticancer effects of everolimus. This novel strategy may serve as an effective and safe approach to GC treatment.

## Supplementary Material

Supplementary figures and tables.

## Figures and Tables

**Table 1:** Correlation between GLUT12 expression and clinicopathological characteristics of 60 GC patients.

Characteristics	Case number	High (*n* = 30)	Low (*n* = 30)	*P* value
Age at surgery (years)				0.605
< 60	32	17	15	
≥60	28	13	15	
Gender				1
Male	44	22	22	
Female	16	8	8	
T stage				0.009*
T1+T2	26	8	18	
T3+T4	34	22	12	
Tumor size (cm)				0.184
< 5	37	16	21	
≥5	23	14	9	
Location				0.602
Cardiac	26	12	14	
Non-cardiac	34	18	16	
TNM stage				0.004*
I+II	31	10	21	
III+IV	29	20	9	
Differentiation				0.197
Poorly	54	29	25	
Well	6	1	5	

* P < 0.05.

## Abbreviations

AR: androgen receptor
ARRIVE: Animal Research: Reporting of *In Vivo* Experiments
CCK-8: cell counting kit-8
CI: confidence interval
COX7C: Cytochrome C Oxidase Subunit 7C
DAPI: 4':6-diamidino-2-phenylindole
DMEM: Dulbecco's modified Eagle's medium
ECAR: extracellular acidification rate
EdU: 5-Ethynyl-2-deoxyuridine
ENO1: enolase 1
FBS: fetal bovine serum
GC: gastric cancer
GEO: Gene Expression Omnibus
GLUT: glucose transporter
GO: Gene Ontology
HK2: Hexokinase 2
HR: hazard ratio
IHC: immunohistochemical
KEGG: Kyoto Encyclopedia of Genes and Genomes
LDHα: Lactate Dehydrogenase α
mTOR: mammalian target of rapamycin
NANS: N-Acetylneuraminate Synthase
NKD-1: NKD Inhibitor Of WNT Signaling Pathway 1
OCR: oxygen consumption rate
OS: overall survival
PBS: phosphate buffered saline
PFS: progression-free survival
PGAM1: Phosphoglycerate Mutase 1
PI: propidium iodide
qRT-PCR: quantitative real-time PCR
SD: standard deviation
shRNA: short hairpin RNA
siRNA: small interfering RNA
SNRNP25: Small Nuclear Ribonucleoprotein U11/U12 Subunit 25
TCGA: The Cancer Genome Atlas
TEAD1: TEA Domain Family Member 1
WB: western blot
YAP1: Yes-associated Protein 1
