# Supplementary material for: Inhibition of androgen receptor enhanced the anticancer effects of everolimus through targeting glucose transporter 12
Source: Int J Biol Sci. 2023 Jan 1;19(1):104–19. doi: 10.7150/ijbs.75106 (PMC9760431; doi:10.7150/ijbs.75106)
Supplement: Supplementary file 1 — Supplementary figures and tables. [file ijbsv19p0104s1.pdf]

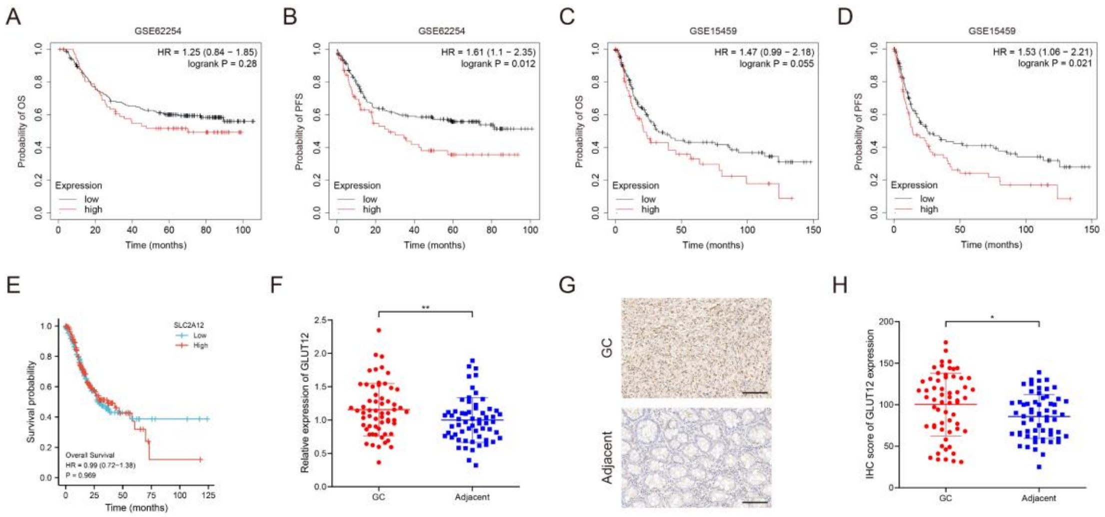

Figure S1. Identification of GLUT12 clinical significance in GC. **A**, **B** The OS (**A**) and PFS (**B**) of GC patients that were divided into the high-GLUT12 group and low-GLUT12 group in GSE62254 dataset. **C**, **D** The OS (**C**) and PFS (**D**) of GC patients that were divided into the high-GLUT12 group and low-GLUT12 group in GSE15459 dataset. **E** The OS of GC patients that were divided into the high-GLUT12 group and low-GLUT12 group in TCGA dataset. **F** The qRT-PCR analysis to show GLUT12 expression in 60 pairs of GC and adjacent normal tissues. **G** The IHC images of one GC case. **H** IHC scores of GC tissues as in (**E**). Scale bar: 100 μm. *$P < 0.05$, **$P < 0.01$.

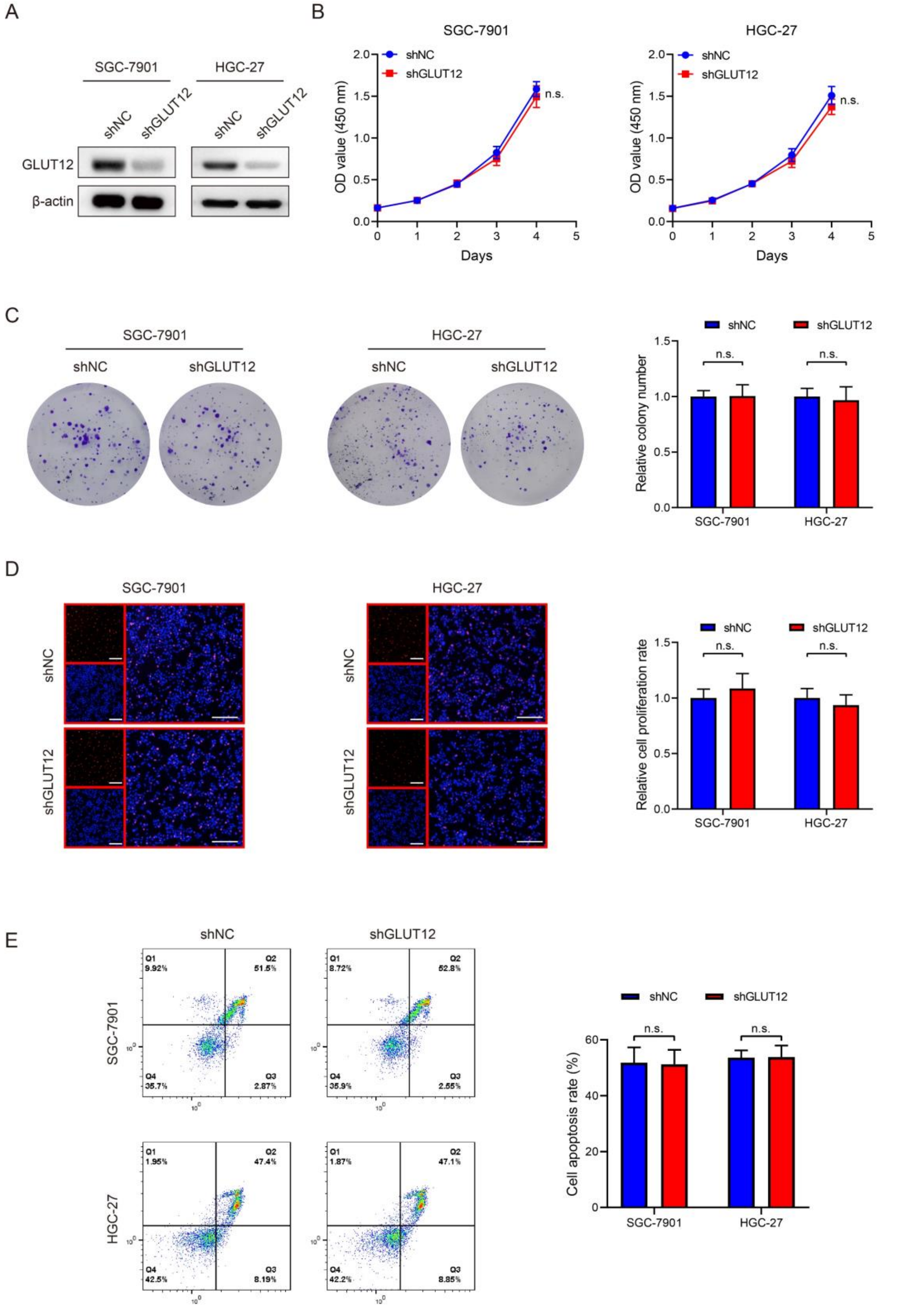

Figure S2. Knockdown of GLUT12 did not significantly attenuate GC proliferation and everolimus resistance. **A** WB analysis to show GLUT12 expression of SGC-7901 and HGC-27 cells that were infected with lentivirus carrying scramble shRNA or shRNA targeting GLUT12. **B-D** The CCK-8

(**B**), colony formation (**C**) and EdU (**D**) assays to measure proliferation of cells as in (**A**). Histograms of colony formation and EdU data are on the right. Scale bar: 100 μm. **E** The flow cytometric analysis to show the apoptosis of cells as in (**A**) that were treated with 10 μM of everolimus. Histograms are on the right. n.s. no significant.

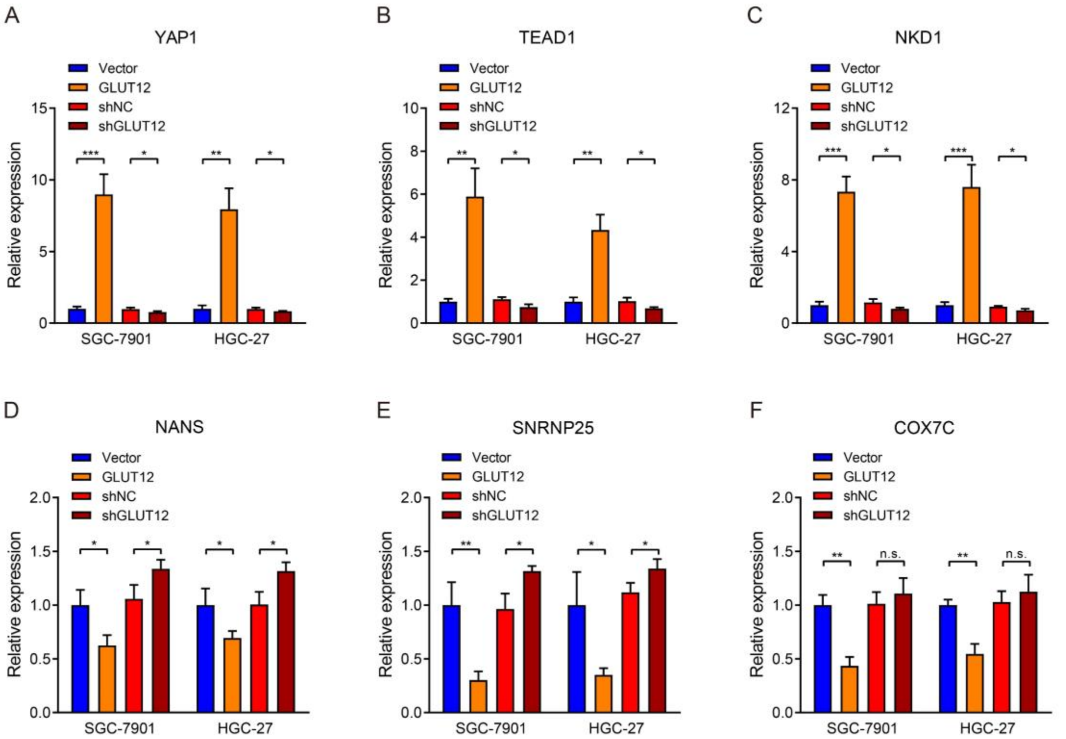

Figure S3. Confirmation of gene expression patterns affected by GLUT12 in GC. **A-F** The qRT-PCR analysis to detect YAP1, TEAD1, NKD1, NANS, SNRNP25 and COX7C expression in SGC-7901 and HGC-27 cells infected with lentivirus carrying empty plasmids or GLUT12 overexpression plasmids or scramble shRNA or shRNA targeting GLUT12. **P* < 0.05, ***P* < 0.01, ****P* < 0.001, n.s. no significant.

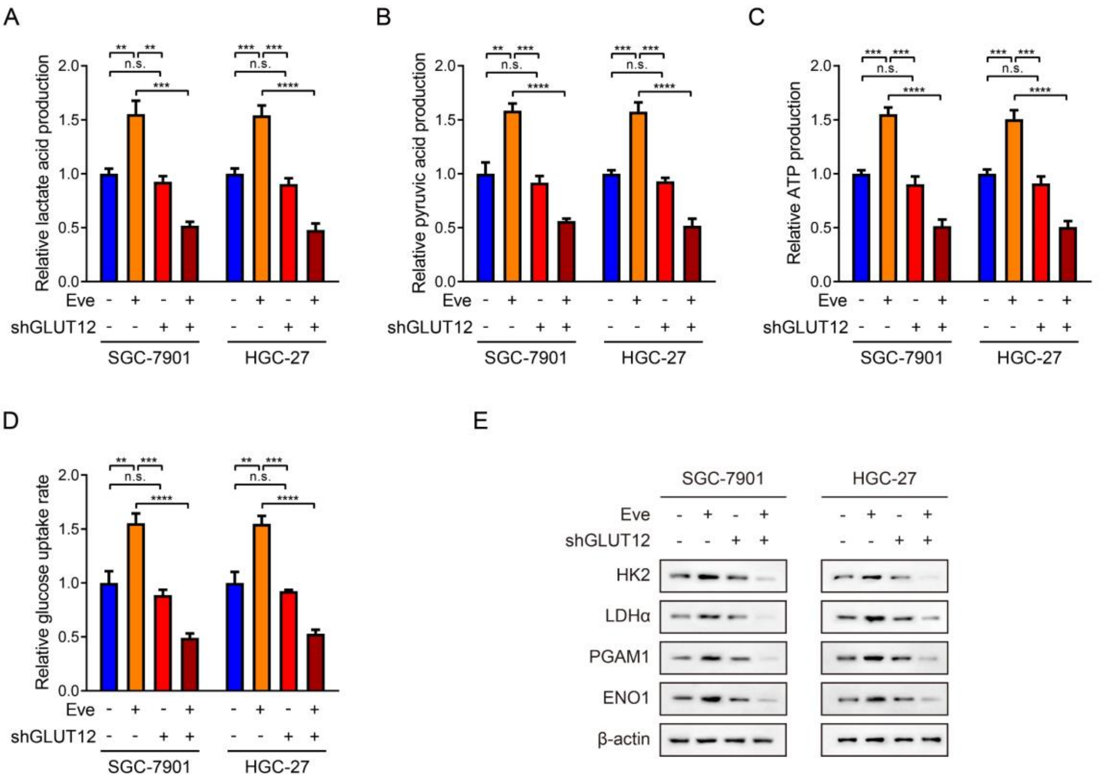

Figure S4. Knockdown of GLUT12 sensitizes GC cells to everolimus and inhibits glycolysis. **A-D** The colorimetric assays to determine the production of lactate acid (**A**), pyruvic acid (**B**), ATP production (**C**) and relative glucose uptake rates (**D**) of SGC-7901 and HGC-27 cells that were treated with vehicle or 5 μM everolimus and infected with lentivirus carrying scramble shRNA or shRNA targeting GLUT12. **E** The WB analysis to show protein expression of cells as in (**A-D**). ***P* < 0.01, ****P* < 0.001, *****P* < 0.0001, n.s. no significant.

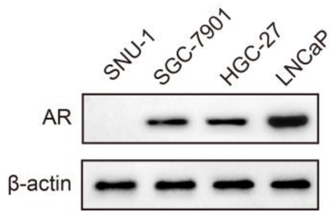

Figure S5. Validation of detectable AR expression in SGC-7901 and HGC-27 cells using WB analysis.

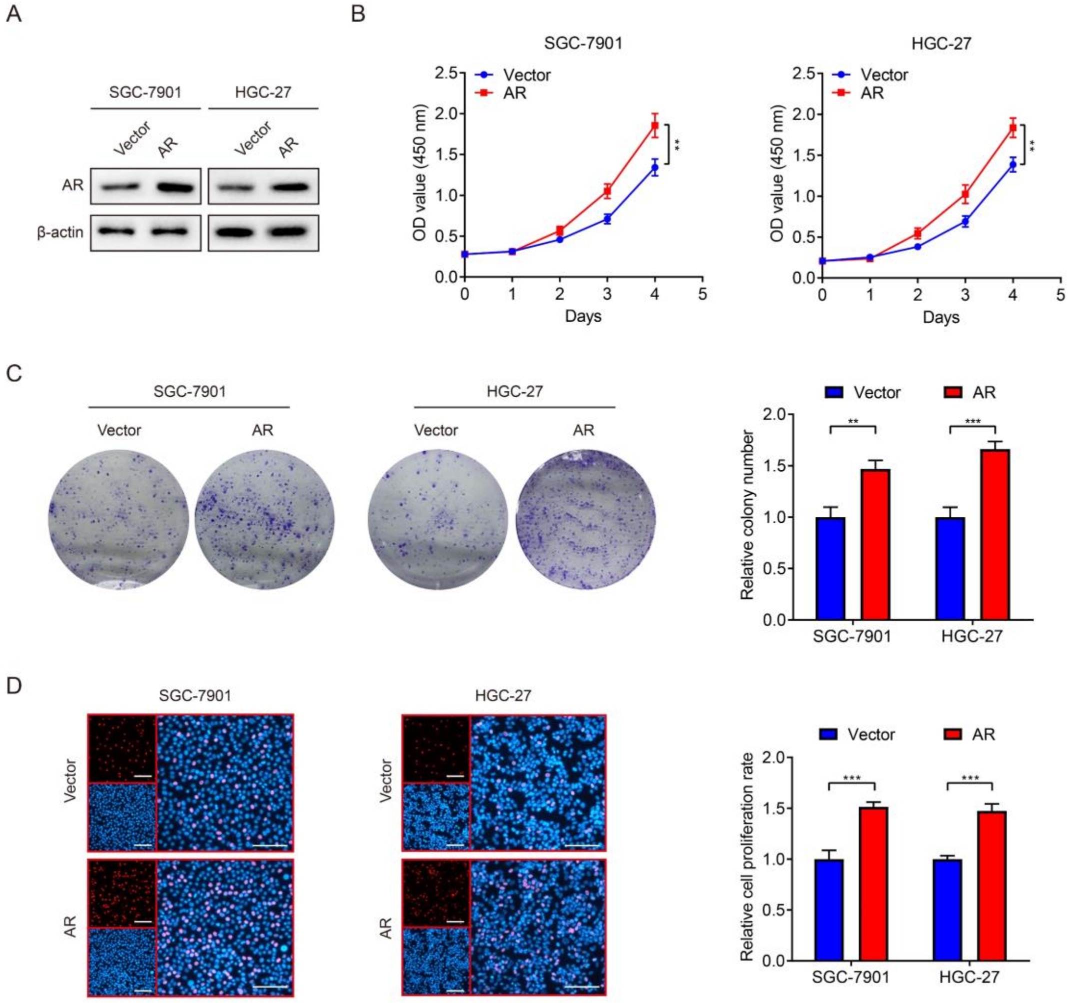

Figure S6. Upregulation of AR expression facilitates GC proliferation. **A** The WB analysis to show AR expression of SGC-7901 and HGC-27 cells that were infected with lentivirus carrying vectors or AR overexpression plasmids. **B-D** The CCK-8 (**B**), colony formation (**C**) and EdU (**D**) assays to measure proliferation of cells as in (**A**). Histograms of colony formation and EdU data are on the right. Scale bar: 100 μm. **P < 0.01, ***P < 0.001.

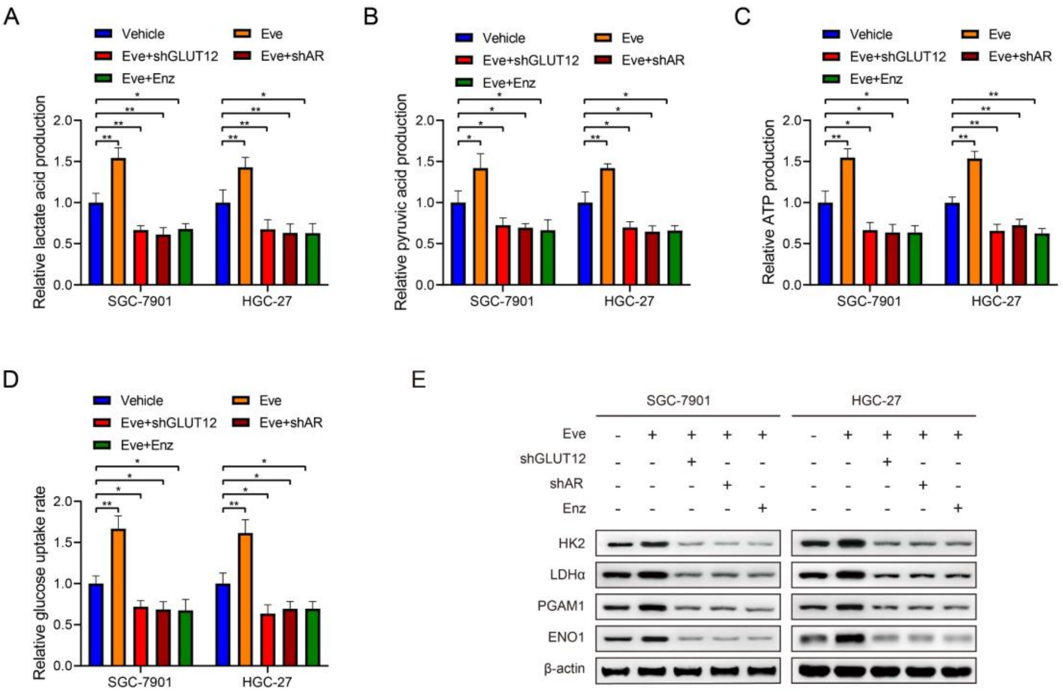

Figure S7. Suppression of AR/GLUT12 pathway inhibits glycolysis activation after everolimus treatment. **A-D** The colorimetric assays to determine the production of lactate acid (**A**), pyruvic acid (**B**), ATP production (**C**) and relative glucose uptake rates (**D**) of SGC-7901 and HGC-27 cells that were treated with vehicle or 5 μM of everolimus. They also underwent control interference, GLUT12 knockdown, AR knockdown or 25 μM of enzalutamide treatment, respectively. **E** WB analysis to show protein expression of cells as in (**A-D**). *$P < 0.05$, **$P < 0.01$.

Table S1. The sequences of oligonucleotides used in this study

Oligonucleotide	Sequence (5'-3')
NC sense	UUCUCCGAACGUGUCACGUTT
NC antisense	ACGUGACACGUUCGGAGAATT
GLUT12 shRNA sense	CAAGGTTCTTGGAAGGTTA
GLUT12 shRNA antisense	TAACCTTCCAAGAACCTTG
AR shRNA sense	GCTGCAAGGTCTTCTTCAA
AR shRNA antisense	TTGAAGAAGACCTTGCAGC
mTOR shRNA sense	CACGGAAGATGTTCGACAA
mTOR shRNA antisense	TTGTCGAACATCTTCCGTG

Table S2. The sequences of primers used in this study

Primer	Sequence (5’-3’)
GLUT1 Forward	ACTCCTCGATCACCTTCTGG
GLUT1 Reverse	ATGGAGCCCAGCAGCAA
GLUT2 Forward	ATCCAAACTGGAAGGAACCC
GLUT2 Reverse	CATGTGCCACACTCACACAA
GLUT3 Forward	GATGGGCTCTTGAACACCTG
GLUT3 Reverse	GACAGCCCATCATCATTTCC
GLUT4 Forward	CCCCAATGTTGTACCCAAAC
GLUT4 Reverse	CTTCCAACAGATAGGCTCCG
GLUT5 Forward	TGACAGCAGCCACGTTGTA
GLUT5 Reverse	GCAACAGGATCAGAGCATGA
GLUT6 Forward	AACATGATGCTCAGCTTCCG
GLUT6 Reverse	CTGACCTGCATCTGACCAAA
GLUT7 Forward	TGTTGTTGATCAGCAGGGTC
GLUT7 Reverse	TGCTGCTTCTATGGTCTTGC
GLUT8 Forward	GAAGCACATGAGAAGCAGCA
GLUT8 Reverse	CTGTGTGCAGCTAATGGTCG
GLUT9 Forward	GGTGCCTGCAATGATGAAG
GLUT9 Reverse	GAGTATCGTGGGCATTCTGG
GLUT10 Forward	CAGCAAAGACACAGAGGCAC
GLUT10 Reverse	GGAAAGTTTGTCCGGCG

GLUT11 Forward	AAACAGGATTGCTGCTGACA
GLUT11 Reverse	CGTGTCTCTGTATCCCCTGG
GLUT12 Forward	ACGAGCCATGGCTTTAACTT
GLUT12 Reverse	CATGGCAGGCCAATAAGAT
GLUT13 Forward	AGCCAGCCATATTGCAAGTC
GLUT13 Reverse	TGTGGCCTACAAATGTTCCA
GLUT14 Forward	ATGGCAAAGATCAGAGCTGG
GLUT14 Reverse	AGGATAGCAGAGAGATGGACAA
YAP1 Forward	CAACTCCAACCAGCAGCAAC
YAP1 Reverse	TTGGTAACTGGCTACGCAGG
TEAD1 Forward	TCCTCACAAGACGTCAAGCC
TEAD1 Reverse	TGCTGCTCGAGAAAAGCTGA
NKD1 Forward	GTCTGGCTGCTACCACCATT
NKD1 Reverse	TGTGGGATGTGGATGGCTTC
NANS Forward	TTGCAGTGTACCAGCGCATA
NANS Reverse	CGCTATGCCTGTTTCATGCC
SNRNP25 Forward	TGTAGTGCAGAGTGCCACAG
SNRNP25 Reverse	CCTCGTCTCGATTCCGGATG
COX7C Forward	AGCATGTTGGGCCAGAGT
COX7C Reverse	ACTGAAAACGGCAAATTCTT
β-actin Forward	CATCCGCAAAGACCTGTACG
β-actin Reverse	CCTGCTTGCTGATCCACATC
